# The Microstructural Evolution of Vacuum Brazed 1Cr18Ni9Ti Using Various Filler Metals

**DOI:** 10.3390/ma10040385

**Published:** 2017-04-05

**Authors:** Yunxia Chen, Haichao Cui, Binfeng Lu, Fenggui Lu

**Affiliations:** 1 School of Mechanical Engineering, Shanghai Dianji University, Shanghai 201306, China; lubf@sdju.edu.cn; 2 Shanghai Key Laboratory of Materials Laser Processing and Modification, Shanghai Jiao Tong University, Shanghai 200240, China; haichaocui@sjtu.edu.cn (H.C.); lfg119@sjtu.edu.cn (F.L.)

**Keywords:** vacuum brazing, filler metal, microstructure, stainless steel

## Abstract

The microstructures and weldability of a brazed joint of 1Cr18Ni9Ti austenitic stainless steel with BNi-2, BNi82CrSiBFe and BMn50NiCuCrCo filler metals in vacuum were investigated. It can be observed that an interdiffusion region existed between the filler metal and the base metal for the brazed joint of Ni-based filler metals. The width of the interdiffusion region was about 10 μm, and the microstructure of the brazed joint of BNi-2 filler metal was dense and free of obvious defects. In the case of the brazed joint of BMn50NiCuCrCo filler metal, there were pits, pores and crack defects in the brazing joint due to insufficient wettability of the filler metal. Crack defects can also be observed in the brazed joint of BNi82CrSiBFe filler metal. Compared with BMn50NiCuCrCo and BNi82CrSiBFe filler metals, BNi-2 filler metal is the best material for 1Cr18Ni9Ti austenitic stainless steel vacuum brazing because of its distinct weldability.

## 1. Introduction

1Cr18Ni9Ti austenitic stainless steel with its fairly good corrosion resistance, creep strength and high temperature mechanical properties is widely used in machinery, nuclear power stations, and chemical, automotive and aerospace applications [1,2,3]. Joining processes, such as welding, brazing and diffusion welding, play an important role in practical applications of these 1Cr18Ni9Ti alloys. Well-developed joining process can not only promote the application of these alloys, but can also provide designers with a versatile choices of materials. Conventional fusion welding processes are often accompanied by high rates of cooling and produced welds with higher ferrite contents and reformed austenite, which result in their poor impact toughness. Brazing is a process in which two or more closely fitting parts are joined via an intermediate metallic material (a brazing metal or alloy) which melts, then the surface being joined is wetted, and it reacts and finally solidifies [4]. Among the various processes, vacuum brazing is one of the most popular methods for 1Cr18Ni9Ti austenitic stainless steel [5,6]. The brazing quality is influenced by many factors including brazing temperature [7], filler metal [8], holding time [9], cooling rate [10], etc. Among these various factors, using a reasonable brazing filler metal is one of the key features of the vacuum brazing process.

The copper, silver, manganese and nickel-based filler metals can be used to braze the nickel alloys and steels. In these filler metals, nickel-based brazing filler metals are primarily used in the cases where extreme heat and corrosion resistance is required. In order to lower the joining temperature, silicon and boron in various concentrations are usually added as melting point depressants. However, boron also has a deleterious effect on the stainless steel brazed joint by forming Cr_3_B precipitates at the base metal, resulting in the lowering of the strength of the brazed assembly [11]. Therefore, silicon and boron appear to have a detrimental effect on the strength of the stainless steel brazed joint. This could possibly be avoided by developing a braze alloy with limited silicon and boron content. Therefore, two commercial fillers for high-temperature application in the brazing of stainless steel were used in this study. They are nickel-based alloy powders, BNi-2 and BNi82CrSiBFe. Additionally, compared with the Ni-base filler metal, the Ni-Cu-Mn alloys makes it more suitable for brazing stainless steel from the viewpoint of the diffusion mobilities [12].

The main objective of this work is to study the reliability of nickel-based filler metal (BNi-2, BNi82CrSiBFe) and manganese-based filler metal (BMn50NiCuCrCo) as filler metals for brazing 1Cr18Ni9Ti tube to 1Cr18Ni9Ti tube (tube-to-tube) by vacuum furnace tests. The microstructures of the brazed joints have been studied using microscopy images. Data on the structure and properties of the joints produced by vacuum brazing using the suggested brazing filler metals are presented. 

## 2. Experimental Procedure

The base metal 1Cr18Ni9Ti austenitic stainless steel used in this study has a nominal composition of Cr 17~19 wt %, Ni 8~11 wt %, Ti 5 wt %, C less than 0.12 wt %, Si less than 1 wt %, Mn less than 2 wt %, the main other element is Fe. Table 1 lists chemical composition of filler metals of two Ni-based alloy powders, BNi-2 and BNi82CrSiBFe, and Mn-based alloy powders, BMn50NiCuCrCo. Before vacuum brazing, the oxides and greasy dirt on the surface of 1Cr18Ni9Ti tube to 1Cr18Ni9Ti tube were removed by sandpaper. The brazing of three filler metals were conducted at 1150, 1150 and 1130 °C respectively for 10 min in a vacuum of 10^−3^ Pa.

The brazed sample was cut by a low speed diamond saw, and followed by a standard metallographic procedure. Microstructures and elemental distribution of the brazed joints were analyzed by means of Hitachi S3400N scanning electron microscopy (SEM) (Hitachi, Tokyo, Japan) equipped with Shimadzu EMPA-1610 Energy-dispersive Spectroscopy (EDS) (Shimadzu, Kyoto, Japan) and HITACHI SU-70 field-emission scanning electron microscope (FE-SEM) with EDS.

## 3. Results and Discussion

### 3.1. Microstructure of the Brazed Joint with BNi-2 Filler Metal

**Figure 1:**
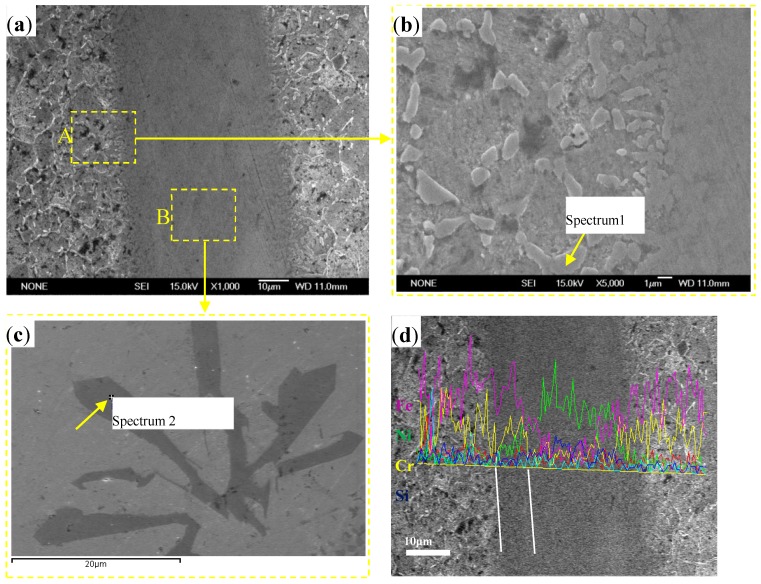
Microstructure of (**a**) brazed joint; (**b**) magnified morphology of zone “A”; (**c**) magnified morphology of zone “B” and (**d**) line scanning analysis.

**Figure 2:**
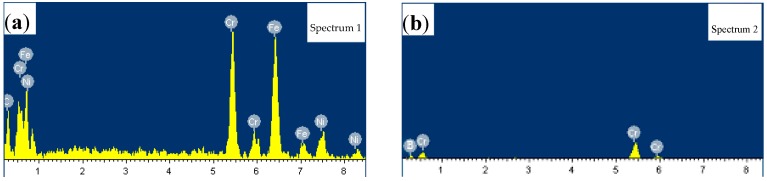
Energy-dispersive Spectroscopy (EDS) results of (**a**) spectrum 1 and (**b**) spectrum 2.

The microstructure of the brazed joint of 1Cr18Ni9Ti steel with BNi-2 filler metal is shown in Figure 1. Chemical composition of the special phases in the brazed region was detected by EDS as shown in Figure 2. The microstructure of the brazed joint is compact and consecutive, no obvious pores or crack defects occurred, as seen in Figure 1a–c which shows that Ni-based alloys had an excellent weldability with 1Cr18Ni9Ti steel. There was a strong diffusion reaction and metallurgical bonds formed between BNi-2 filler metal and base metal.

In Figure 1b, there is a chain of small shapeless precipitation phases formed in the austenite grain boundaries near the brazed region. The EDS result (see in Figure 2a) indicates that the precipitation phase is mainly composed of C, Cr, Ni and Fe elements. Therefore, precipitation phases were considered to form during brazing, and the Fe element was determined to be diffused from the base metal.

Elemental distribution across the brazed region of 1Cr18Ni9Ti steel with BNi-2 filler metal was measured by EDS and the result is shown in Figure 1d. Fe and Cr diffused from 1Cr18Ni9Ti steel to the brazed region while Ni and Si diffused in the opposite direction. The elemental content of Cr and Fe about 10 μm near the base metal are higher than that of the brazed region and lower than that of the base metal, while Ni and Si are lower than that of the brazed region and higher than that of the base metal. Therefore, there was an interdiffusion region (width about 10 μm) consisting of Fe, Cr, Ni and Si in the diffusion affected zone of 1Cr18Ni9Ti steel.

In addition, some precipitation phases were formed in the brazed region (spectrum 2 in Figure 1c). The EDS analysis results presented in Figure 2b indicate that the precipitation phase is mainly composed of B and Cr elements and it is identified as CrB and Cr_3_B phases which exist in the braze alloy after vacuum brazing. However, Boron is a very small atom, and its diffusivity is much higher than that of Si, Cr, Ni and Fe atoms with a larger atomic radius. It will cause nonsymmetrical mass transport during interdiffusion. Some Kirkendall porosity close to the braze will be formed if the rate of interdiffusion is not balanced [13]. Therefore, it is noted that the amount of CrB and Cr_3_B phases in the joint should be kept at a reasonable level.

### 3.2. Microstructure of the Brazed Joint with BNi82CrSiBFe Filler Metal

**Figure 3:**
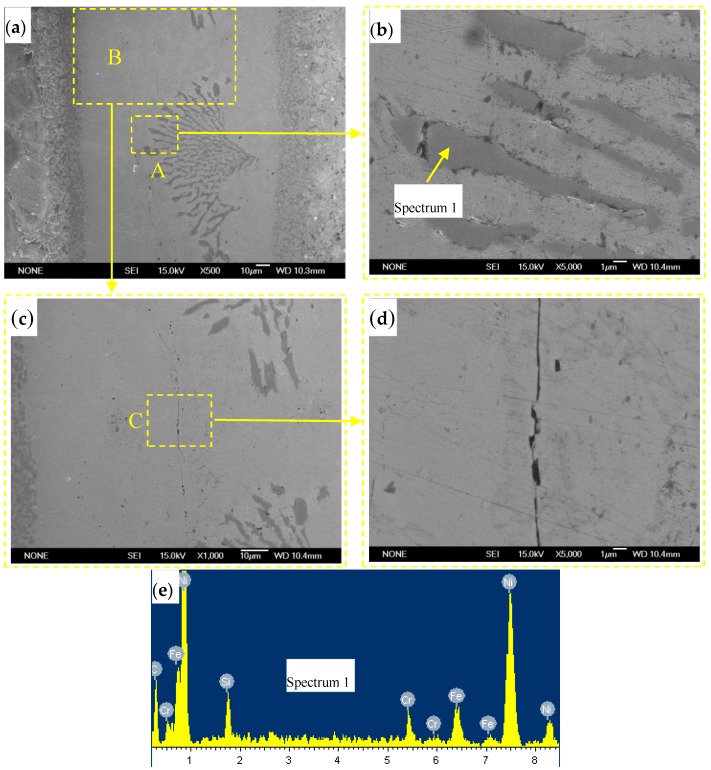

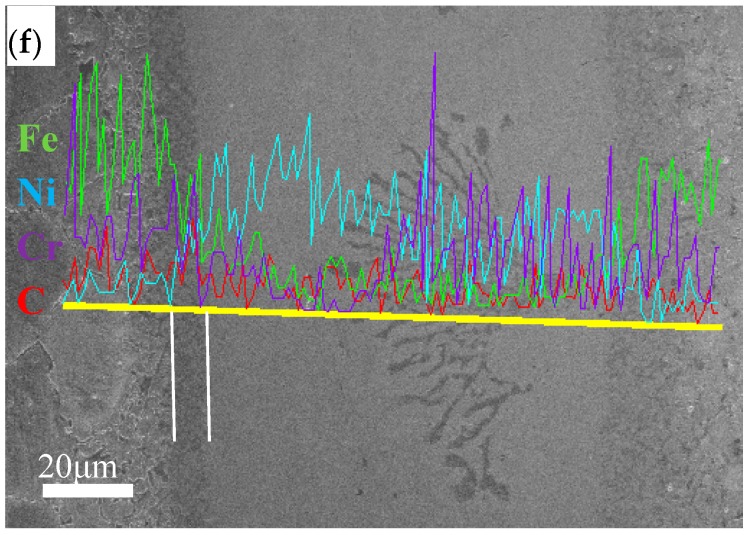
Microstructure of vacuum brazing joint of 1Cr18Ni9Ti with BNi82CrSiBFe filler metal: (**a**) brazed joint; (**b**) magnified morphology of zone “A”; (**c**) magnified morphology of zone “B”; (**d**) magnified morphology of zone “C”; (**e**) EDS results of spectrum 3 and (**f**) line scanning analysis.

Compared with BNi-2 filler metal, BNi82CrSiBFe filler metal contained more Si and B, and less Fe. Figure 3a–d shows a microstructure of the brazed joint of 1Cr18Ni9Ti steel with BNi82CrSiBFe filler metal. Chemical composition of the special phases in the brazed region was detected by EDS, as shown in Figure 3e–f. A considerable number of strip blocks and cracks can be observed in the middle of the brazing region in Figure 3a. The EDS analysis results (spectrum 1 in Figure 3b) indicate that the strip blocks are mainly composed of C, Cr, Ni and Fe elements. Therefore, the precipitation phases are identified as (Cr, Ni, Fe)C carbides formed during brazing solidification. Cracks appear close to precipitation phases and propagate along longitudinal direction of the brazing joint in Figure 3c,d. The possible reason for this is that new (Cr, Ni, Fe)C carbides may result in the content of Cr, Ni remarkable decrease and cause the solidification cracks to occur.

Figure 3f shows the results of linear SEM-EDS of the joint. There was also an interfacial reaction region (width about 10 μm) formed by cooling of the bonded specimen prior to the completion of brazed center solidification, which underwent a thermal solidification process. However, this interdiffusion region consists of Fe, Cr, Ni and C elements which easily form new brittle (Cr, Ni, Fe)C carbides. The weldability of BNi82CrSiBFe filler metal exhibiting on the base metal is not as good as that of BNi-2.

### 3.3. Microstructure of the Brazed Joint with BMn50NiCuCrCo Filler Metal

**Figure 4:**
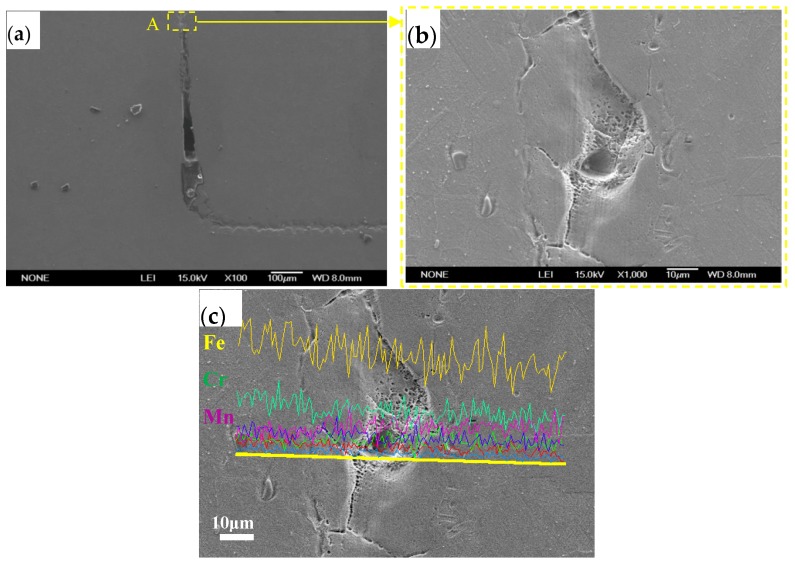
Microstructure of vacuum brazing joint of 1Cr18Ni9Ti with Mn-based filler metal: (**a**) the center of brazed joint, (**b**) magnified morphology of zone “A” and (**c**) line scanning analysis.

Figure 4 shows the microstructure of the center (filler alloy) of the brazed joint of 1Cr18Ni9Ti steel with BMn50NiCuCrCo filler metal. Defects like pits, pores and cracks can be observed in Figure 4b. The EDS analysis results presented in Figure 4c indicate that there was little change in the elemental contents of Fe, Cr and Mn along the transverse direction of the brazing joint. In other words, the element’s interdiffusion region does not exist in this brazed joint. Therefore, the joint strength of the brazing joint became worse. Due to the Mn-based filler metal having a limited spreadability and insufficient wettability on the base metal during brazing, the base metal cannot be completely filled with the molten Mn-based filler metal. Thus, pits, pores and crack defects will form after the brazing joint finishes solidifying [14].

## 4. Conclusions

The vacuum brazing of 1Cr18Ni9Ti austenitic stainless steel tube–tube structures with nickel-based filler metal (BNi-2, BNi82CrSiBFe) and manganese-based filler metal (BMn50NiCuCrCo) has been performed in this paper. The effect of per filler metal on microstructure and weldability has been discussed. The following conclusions can be drawn:

(1) It is feasible to braze 1Cr18Ni9Ti tube–tube structures using BNi-2 as brazing filler. Microstructural results show that the microstructure of the brazing region is compact and free of porosity or crack defects. Due to good weldability of BNi-2 on the base metal, there is an interdiffusion region (consisting of Fe, Cr, Ni and Si and its width about 10 μm) close to the base metal. In addition, it is confirmed that CrB and Cr_3_B boride phases formed in brazing were helpful to prevent porosity or crack defects.
(2) Through microstructural observation there is an interdiffusion region (consisting of Fe, Cr, Ni and C and its width about 10 μm) close to the base metal and a considerable amount of strip blocks and cracks in the brazing region with *BNi82CrSi*BFe filler metal. However, new (Cr, Ni, Fe)C carbides may result in the content of Cr, Ni remarkable decrease and cause the solidification cracks to occur.
(3) The microstructure of the brazed joint with BMn50NiCuCrCo filler metal indicates that the Mn-based brazing filler had a poor weldability on the base metal. As a result of poor weldability, there is no interdiffusion region but defects such as pits, pores and cracks existed in the brazing joint.

## Figures and Tables

**Table 1:** Chemical composition of three types of brazing filler metals (in weight percent).

Filler Metal	Element												Melting Temperature Solids Liquids		Braze Temperature (Approx.) °C
	Ni	Cr	Fe	B	Si	Co	Al	Mn	Mo	Ti	Cu	C			
BNi-2	86.63	7.47	3.24	2.58	0.038	0.0232	0.015	0.0067	0.003	0.0034	–	0.06	970	1000	1150
*BNi82CrSi*BFe	Bal.	6–8	–	3–3.5	4–5	–	–	–	–	–	–	0.06	970	1000	1150
*BMn50NiCuCrCo*	27	4.5	–	–	–	4.5	–	Bal.	–	–	13.5	–	1010	1035	1130
